# “I’m learning to live after cancer and its treatment”: Exploring the challenges of cancer survivorship in Arkansas

**DOI:** 10.1016/j.ssmqr.2024.100519

**Published:** 2024-12-19

**Authors:** Ramey Moore, Emily Hallgren, Shani Worrell, Anna Wahls, Sarah K. Council, Deanne L. King, Rajalakshmi Cheerla, Donya Watson, Jacquelene Childs, Martha Garrett-Shaver, Pearl A. McElfish

**Affiliations:** a College of Medicine, University of Arkansas for Medical Sciences Northwest, 2708 S. 48th St., Springdale, AR, 72762, USA; b Institute for Community Health Innovation, University of Arkansas for Medical Sciences Northwest, 2708 S. 48th St., Springdale, AR, 72762, USA; c Department of Otolaryngology, University of Arkansas for Medical Sciences, 4301 W. Markham St., #543, Little Rock, AR, 72205, USA; d College of Medicine, University of Arkansas for Medical Sciences, 4301 W. Markham St., #530, Little Rock, AR, 72205, USA; e South Arkansas Regional Hospital, 706 West Grove St., El Dorado, AR, 71730, USA

**Keywords:** Rural, Survivorship, Psychosocial, Physical functioning, Theory-driven qualitative research, Quality of life

## Abstract

**Purpose:**

This study explores challenges faced by a large, diverse sample of cancer survivors who received care in the rural state of Arkansas to provide holistic insight into the quality of survivorship.

**Methods:**

We used a theory-driven exploratory descriptive design to explore cancer survivors’ (n = 519) biggest cancer-related challenges.

**Results:**

Cancer survivors’ challenges were organized using *a priori* domains from the Quality of Survival framework: quality of life, survival, managing the side effects, and managing the economic impact. Nearly half (48.4%) of all responses identified challenges related to quality of life, and 39.7% of all responses focused on the challenges of managing the side effects of cancer and its treatment. Managing the economic impact (6.9%) and survival (4.9%) were also identified as challenges by survivors.

**Conclusions:**

Survivors faced a range of challenges from cancer and its treatment. Survivors primarily faced psychosocial challenges, issues with ongoing care, and physical and/or functional sequelae. Managing the economic impact and financial toxicity of cancer treatment and surviving cancer and its treatment were less frequently identified among the challenges faced by survivors.

**Implications for cancer survivors:**

Our analysis highlights the importance of understanding the psychosocial and physical sequelae of cancer and its treatment. Our research helps fill significant gaps in the literature, improving the understanding of rural cancer survivorship. Nuanced understanding of survivors’ experiences, using theory-driven qualitative and mixed-methods approaches, will continue to be critical for developing effective, evidence-based practices to meet the needs of survivors.

## 1. Introduction

Millions of people in the United States (US) and around the world live beyond their cancer diagnosis and treatment (Fallowfield et al., 2017), and the number of cancer survivors in the US is projected to reach 22.2 million by 2030 (American Cancer Society, n.d.; Gallicchio et al., 2021; Jung, Kay, Robinson, Sheppard, & Mayer, 2022). The substantial increase in cancer survivors has been attributed to improvements in cancer screening, diagnosis, treatment, supportive care, and an aging US population (American Society of Clinical Oncology’s Cancer.Net, 2021; Gallicchio et al., 2021; Jung et al., 2022). Today, 69% of survivors live five years or longer beyond their diagnosis, 47% live 10 or more years beyond their diagnosis, and 18% are living 20 years or more (Gallicchio, Devasia, Tonorezos, Mollica, & Mariotto, 2022). Given the large and increasing number of cancer survivors, it is important to understand their challenges and needs (Gallicchio et al., 2021; Jung et al., 2022; National Research Council, 2006). Prior research has documented that cancer survivors report a range of challenges, including the burden of cancer symptoms, side effects arising from treatment, diminished quality of life, and financial toxicity, to say nothing of the effects on caregivers, families, and the survivor’s social relationships (Fallowfield et al., 2017; Gilbert, Miller, Hollenbeck, Montie, & Wei, 2008; Han, Robinson, Jensen, Smith, & Yabroff, 2021; Hewitt, Greenfield, & Stovall, 2005).

However, research on cancer survivorship is limited, and there remains a critical gap in our knowledge regarding the unique challenges facing cancer survivors. In 2019, the National Cancer Institute identified several critical evidence gaps in cancer survivorship research, including the psychosocial effects and sequelae of cancer and its treatment (Gallicchio et al., 2021). Other critical evidence gaps include a comprehensive understanding of the range of social needs, e.g., social support, financial impact, and access to care, which must be met to provide evidence-based, high-quality survivorship care for the rapidly increasing population of cancer survivors in the US (American Cancer Society, n.d.; Gallicchio et al., 2021; National Research Council, 2006). Methodologically, there is limited research using qualitative methods to develop in-depth understandings of survivors’ challenges and needs and how best to address them. Other gaps in the literature include the challenges of managing cancer care in rural communities, which often face significant cancer health disparities including access to care, transportation, participation in screening, and diagnosis at advanced stages, among others (Adams et al., 2017; Andrilla, Moore, Man Wong, & Evans, 2020; Bhatia et al., 2022; Blake, Moss, Gaysynsky, Srinivasan, & Croyle, 2017; Hallgren et al., 2020; Hallgren et al., 2023; Hallgren, Yeary, et al., 2023; Petermann et al., 2022; Segel & Lengerich, 2020; Yabroff, Han, Zhao, Nogueira, & Jemal, 2020). Prior studies have explored quality of life among cancer survivors, but are often broad in scope or focused on a small number of measures, e.g., physical functioning, pain, and mobility (Jacobsen & Jim, 2011). Additional critiques include recent research by Thompson et al. (2024) noting that the concept of survivorship is currently under-theorized, with few studies integrating theoretical frameworks to facilitate holistic understanding of the challenges faced by survivors (Thompson et al., 2024).

### 1.1. Quality of survival

The current study uses the Quality of Survival (QoS) framework (Fallowfield et al., 2017). Fallowfield et al. (2017) have proposed the QoS framework which provides a holistic, patient-centered perspective on cancer survivorship with a focus on the quality of a survivor’s life (Fallowfield et al., 2017). The QoS framework was developed through a multi-stage and multi-stakeholder process, including interviews with patients with two cancer types (metastatic non-small cell lung cancer and metastatic melanoma) in the US and Canada, oncologists (in community and academic settings), and representatives of payers in the US, UK, France, and Germany (Fallowfield et al., 2017).

The QoS framework includes four interconnected and overlapping domains: quality of life, survival, managing the side effects, and managing the economic impact (Fallowfield et al., 2017). *Quality of life* refers broadly to a survivor’s well-being, including life satisfaction or self-esteem (Cella & Stone, 2015; Fallowfield, 2009; Fallowfield et al., 2017). *Survival* refers to the length of a survivor’s life since diagnosis with cancer and the related psychosocial meanings and experiences of survival (e.g., extending one’s life, being declared cancer-free, etc.) (American Society of Clinical Oncology’s Cancer.Net, 2021; Shrestha et al., 2019). *Managing the side effects* includes the diverse biopsychosocial and therapeutic responses to the range of sequelae and side effects experienced by survivors, including functional changes affecting everyday life (Croghan, Cullen, & Raheem, 2023; Fallowfield et al., 2017). *Managing the economic impact* includes the financial and economic effects of survivorship, whether directly or indirectly related to cancer and its treatment (Fallowfield et al., 2017). While the QoS exhibits potential as a framework to understand how survivors can best survive and thrive beyond cancer, to the authors’ knowledge, no studies have applied the QoS in real-world research settings or with large samples of diverse survivors with a range of cancer types. Thus, the framework and its domains may require elaboration and operationalization in real-world settings. This is especially critical due to the marked heterogeneity in extant measures of patient-reported outcomes in survivorship which renders comparison across studies difficult, and the relative dearth of broadly-generalizable, holistic qualitative measures (Pangestu & Rencz, 2023).

In light of the gaps in the cancer survivorship literature, this study explores the challenges of a large, diverse sample of cancer survivors who received cancer care in the rural state of Arkansas. The challenges are expressed in cancer survivors’ own words, leveraging the QoS framework to guide our analysis. The purposes of this study are, first, to contribute to the qualitative literature describing the challenges of cancer survivors, particularly those living in states such as Arkansas with significant rural populations who have been under-studied and under-resourced, and second, to provide the first real-world application of the QoS framework to a large, diverse sample in order to explore its effectiveness as a potential framework for collecting and evaluating holistic qualitative outcomes during cancer survivorship.

## 2. Methods

### 2.1. Study design

This study used a theory-driven exploratory descriptive qualitative design to explore cancer survivors’ biggest challenges related to their cancer (Colorafi & Evans, 2016; Hunter & McCallum, 2019). All study materials and procedures were reviewed and approved by the University of Arkansas for Medical Sciences Institutional Review Board (IRB #274075).

### 2.2. Participant recruitment and study sample

The study team used electronic medical record (EMR) abstraction from the University of Arkansas for Medical Sciences to identify potential participants for a cancer survivorship survey. Inclusion criteria included receipt of a cancer diagnosis or treatment for cancer within the past 3 years and being 18 years of age or older at the time of diagnosis or treatment. Patients who received a cancer diagnosis within the past six months were excluded from recruitment to reduce patient burden. Study staff sent study invitations by mail, email, and/or SMS to eligible patients between September 2022 and August 2023. Survivors could complete the survey by mail, online using REDCap, or over the phone. Survivors received a description of the study, which included an explanation of the estimated time for completion of the survey, potential risks and benefits, voluntary nature of participation, and confidentiality of their responses. Completion of the survey indicated consent to participate. Survivors who completed the survey were provided a $40 gift card.

**Fig. 1.**
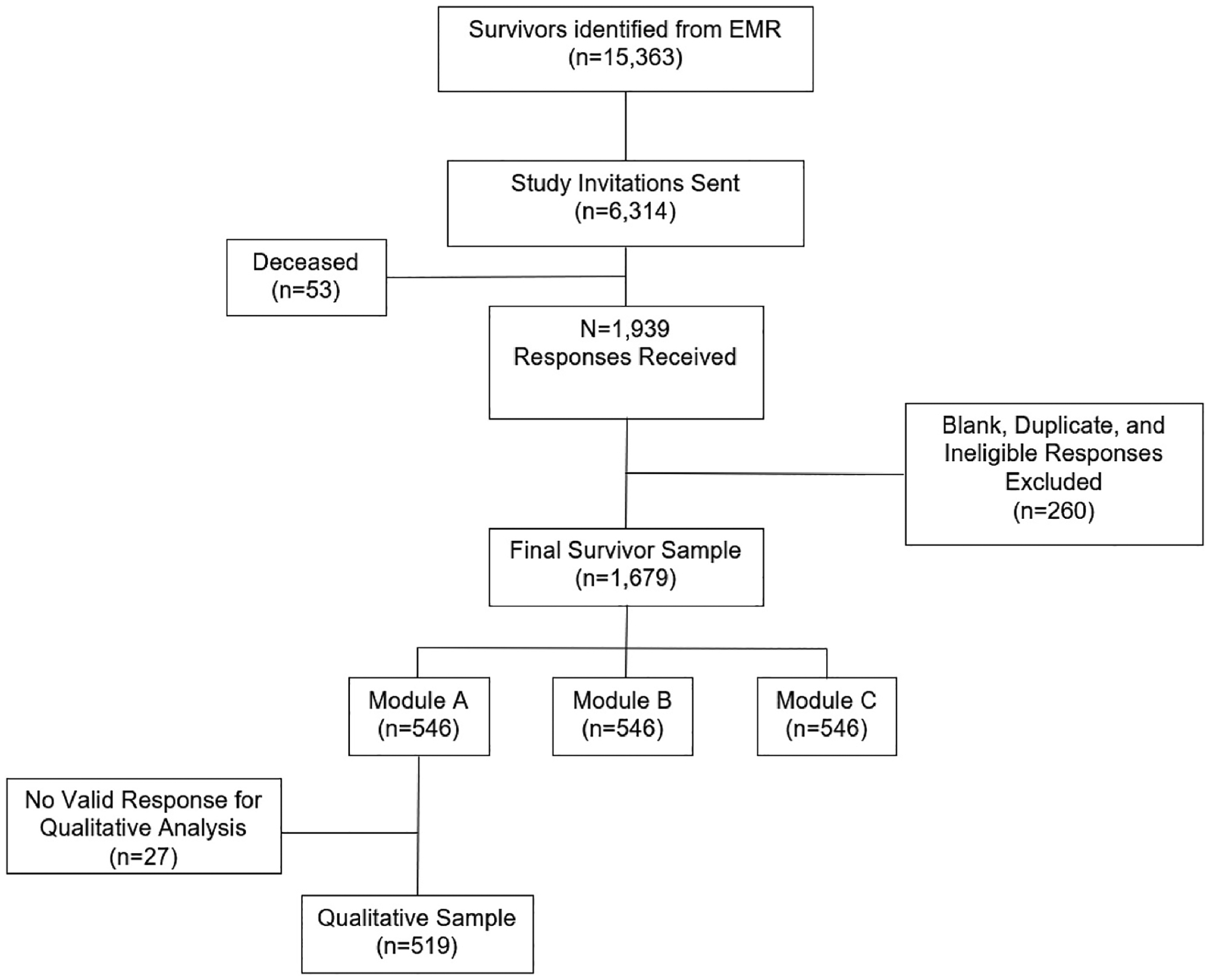
Study Sample Flowchart EMR = electronic medical record.

The survey instrument was developed by the study team with guidance from a physician advisory group, oncology social workers, and a community advisory board that included cancer survivors and caregivers. The survey was available in English and Spanish. To reduce participant burden, the survey instrument contained a set of core questions and three different randomly-assigned modules. The present study analyzes responses to Module A (n = 546). A total of 519 of these survey participants responded to the open-ended question analyzed in this study, and these participants comprise the analytical sample for this study. Fig. 1 presents the study enrollment and steps used to generate the analytical sample for this study.

### 2.3. Data analysis

Demographic information was collected using items from the Behavioral Risk Factor Surveillance System and Health Information National Trends Survey (Centers for Disease Control and Prevention (CDC), 2021; Nelson et al., 2004). To assess cancer-related challenges experienced by survivors, we asked, “In your own words, what is the biggest challenge you’re currently facing related to your cancer?” This broad question aligns with the exploratory descriptive paradigm for the study, providing space for survivors to present their lived experience while minimizing the potential effects of bias during data collection (Creswell & Creswell, 2018).

Survivor sociodemographic characteristics are provided in Table 1. We used MAXQDA 2020 to systematically organize survivor responses, identify patterns in meanings, and generate code frequencies (Kuckartz & Kuckartz, 2002; U. Kuckartz & Rädiker, 2021). We used the QoS framework to structure analysis and coding (Fallowfield et al., 2017). The QoS is comprised of four domains: quality of life, survival, managing the side effects, and managing the economic impact (Fallowfield et al., 2017). We used QoS domains as *a priori* primary codes and to organize emergent codes during analysis. The lead author developed an initial codebook, which was then reviewed by three co-authors with qualitative research expertise. We used an iterative process to revise the codebook. An initial review of the codebook was conducted by two co-authors. The codebook was revised three times prior to final coding of the dataset. Confirmation coding was conducted by two co-authors with all discrepancies in coding resolved using a consensus approach where all coders discussed and reached unanimous agreement on all coded segments. Parsimony of coding was a primary focus during this process as the study team encountered significantly under-elaborated or poorly operationalized constructs within this framework, but also within the extant literature on survivorship and among patient-reported outcomes and measures, broadly. We elaborate on this issue below, although a full exploration of these issues is outside the scope of the present study.

At the completion of the coding process, we used MAXQDA 2020 to calculate the frequencies of each code across all coded segments. The study team classified participants using Rural-Urban Continuum Codes (RUCC) based on their county of residence (Moss et al., 2021; United States Department of Agriculture Economic Research Service, 2024). Following the RUCC schema, counties with RUCC codes 1–3 were designated as metropolitan (i.e., urban), while codes 4–9 were categorized as nonmetropolitan (i.e., rural). This categorization allowed for comparative analysis of coded segment frequencies between participants living in urban and rural areas in the qualitative dataset. Using a confirmatory approach, we also used MAXQDA to generate code frequencies organized by their residence, which we report below. As survivors’ statements were complex and often included multiple domains in the same statement, we present only the most representative quotes within each code below.

## 3. Results

Table 1 presents the sociodemographics of our analytical sample (n = 519). All study participants were cancer survivors whose ages ranged from 21 to 95 years of age, with a mean age of 63 years. Women made up more than half of the study sample (60.2%). The majority of the sample identified as White (80.9%), with the second largest category being Black or African American (14.5%). The majority reported they were married or co-habitating (62.1%). Almost half were retired (43.4%), over a quarter were employed (28.8%), and more than half (64.2%) had at least some college experience. Most survivors (98.4%) reported having some form of insurance coverage. About 40% of the sample resided in rural areas, and about 60% resided in urban areas, which is consistent with the most current census data for the state (McElfish et al., 2023; United States Census Bureau, 2024). A small number of participants (5.6%) resided outside the state of Arkansas at the time of survey completion, but may have resided in the state at some point during their diagnosis and/or treatment. The sample was diverse in cancer sites and/or type. See Table 2.

The QoS framework identifies four broad, interconnected domains which we used as *a priori* primary codes. These four QoS domains include: *quality of life*, *survival*, *managing the side effects*, and *managing the economic impact*. We used the QoS domains to organize emergent sub-codes. Table 3 presents the final codebook (*a priori* and emergent sub-codes) and the frequency of each code across all coded segments, as well as the proportion of the sample who identified one or more challenges in each domain. Due to the nature of qualitative research, survivors’ responses frequently included multiple QoS domains and/or sub-codes in their responses.

### 3.1. Quality of life

Nearly half (48.4%) of all coded responses mentioned quality of life and related factors in describing their greatest challenges, and 78.6% of survivors described at least one challenge affecting their quality of life. The most frequent sub-code within quality of life was for the psychological and emotional effects of cancer and its treatment (28.2%). Survivors also described social challenges resulting from their cancer and treatment (5.4%), as well as issues related to their experiences with ongoing and/or follow-up care (14.8%).

#### 3.1.1. Psychological/emotional

Survivors frequently identified a range of psychological and emotional challenges stemming from their cancer and/or its treatment. One survivor described their cancer’s toll on their quality of life, stating, “It has changed me completely; I’m no longer the happy person I once was; I do not like [how] it makes me feel emotionally, physically” (PID 2475). Another survivor identified the struggle to maintain a positive outlook, explaining that their biggest cancer-related challenge was to “[make] myself to be positive” (PID 4974). One survivor linked not being “mentally capable of doing things I used to” to feelings of “extreme anxiety” as their biggest challenge (PID 3910).

Some survivors identified emotional challenges arising from their cancer treatment, both directly and indirectly. One survivor directly linked their emotional challenge to the physical effects and outcomes of their treatment: “My implant and natural breast are no longer symmetrical which hurts my self-esteem” (PID 3010). Another survivor’s greatest challenge related their social identity as a cancer survivor to feelings of guilt and shame arising from not having received chemotherapy or radiation therapy for their cancer, stating, “I’m so grateful I didn’t have to do chemo or radiation but I feel ashamed when I have to tell people I didn’t have to do that when all of the people I know that have breast cancer had to have all of those treatments. It makes me feel very guilty” (PID 1303).

Another challenge facing many survivors was the fear of recurrence of their cancer, its spread to other body systems or sites, or diagnosis of another form of cancer. One survivor succinctly described their greatest challenge as “worrying the cancer will come back” (PID 248). These fears could have a scope which extended beyond a survivor’s individual cancer returning, as one survivor stated: “I’m always afraid of cancer coming back or any other [cancer] for that matter” (PID 370). Fears of recurrence were also complex. One survivor stated, “I constantly worry that it will come back, and that when it does come back it will be worse than before. I think about this several times a week and get super anxious about it regularly, especially when I’m not feeling well” (PID 5252).

#### 3.1.2. Social

Survivors also described social challenges. One survivor noted reduced capacity for engaging in activities they enjoy, writing, “Not being able to do activities or social functions as I did before or crafts” (PID 4041). Survivors described difficulty “being in social settings at times with my anxiety” (PID 2593). They also described changes to their social lives. One survivor identified their greatest challenge as “the people that know I do not have breast” (PID 4232). Another survivor included the challenge of being “embarrassed with the [facial] scar [because] they had trouble closing the surgery site” (PID 3267).

Other survivors noted intimate relationships were affected by their cancer or its treatment. One survivor recounted difficulty “being intimate with a partner since having my ostomy surgery that led to a permanent colostomy bag and urostomy bag” (PID 2593). Another survivor faced a similar challenge “keeping from leakage and clothes odor when I leak even a little otherwise normal life. No girlfriend, no sexual activity, or miss loving a dating someone the most” (PID 75).

These challenges were described as affecting several aspects of their social life at the same time. One survivor summarized their greatest challenge as being “unable to be social or hold a job” (PID 3032). Another survivor noted, “the side effects [of my chemotherapy drugs] are so debilitating that I cannot properly attend to my business, help my wife with things at home and be unable to play my tuba in the local community band” (PID 3225). Survivors presented a complex picture of the social challenges associated with their cancer, with one survivor stating, “Cancer has robbed me of my energy, income and sometimes, happiness. We wanted to travel and enjoy life but now we can’t … It’s robbed me of my dignity” (PID 2660).

#### 3.1.3. Ongoing/follow-up care

Cancer survivors also identified challenges directly related to their ongoing and follow-up cancer care. Survivors reported increased stress or anxiety related to scans and other follow-up care. One survivor described their biggest challenge as “the concerns surrounding reoccurrence,” manifesting as “the stress level I feel prior to a check-up is incredible” (PID 46). While closely linked to fear of recurrence, many survivors similarly linked their challenges to feelings intensified by continuing with monitoring as recommended by their providers. One survivor described “making my appointments” for annual mammograms and follow-up care with her “breast doctor” as her primary challenge which she linked to delaying or avoiding her appointments (PID 1650). Dealing with daily preventive care was also a challenge, as one survivor stated, “having to take medications everyday for the rest of my life” (PID 2916).

Issues with their care team or the delivery of care were also described as challenges. One survivor described her greatest challenge as:

Getting to see my doctor. My doctor has changed 3 times and I have not like that. I am a nurse practitioner but I make an appointment to see my doctor but just prior to my appointment, someone switches me to a nurse practitioner. I missed seeing my oncologist in 2022 because of this and it has made me very sad. I am driving a few hundred miles to see my oncologist. Why can’t I see him? I love practitioners. I am one. But I should decide if I want a non-oncologist to monitor me. There may come a time when I’m ready. I’m not now. I wish someone could help me.

(PID 2181)

Survivors also described challenges in finding an oncologist who met their individual needs and/or in developing a satisfactory therapeutic relationship with their oncologist. One survivor stated her greatest challenge was “never finding a gynecological oncologist that truly knew anything about treating me” (PID 2162). Communication with one’s care team was also noted as a challenge. One survivor responded that their challenge was “lack of thorough communication with doctors, [and after an unsuccessful surgery], how I was treated when I communicated about this” (PID 5200). Some survivors also noted that their cancer treatment becomes challenging in the context of their overall health and comorbidities. Describing their experience, one survivor wrote that “due to having a heart attack in September 2022, my largest challenge now is how to balance my cancer diagnosis and treatment with my new heart condition and treatment” (PID 4645).

### 3.2. Survival

Only 8.1% of survivors and 4.9% of coded segments described surviving their cancer or its treatment as a challenge, which was the least represented domain of the QoS framework. One survivor articulated this challenge as the “fear of not making it” (PID 4431). Another described the challenge of “knowing I am/was gonna die, [and] never see my son” (PID 2631). The impact of one’s survival on family was a closely-related challenge. One survivor wrote that their biggest challenge was the “worry about dying leaving my family” (PID 4993). Some survivors answered with positive descriptions of their challenges related to survival, especially the extension of their lifespan. One survivor wrote, “Trying to live + eat as healthy as I can so that I’m doing all I can to keep the cancer from coming back! My mom died at 54 from metastatic breast cancer, so I want to beat that age!” (PID 3605). Reaching some form of survival milestone was also echoed by a survivor whose challenge was “reaching my 100th B-Day” (PID 2881).

### 3.3. Managing the side effects

The management of side effects during and after active treatment, e.g., pain, fatigue, and short- or long-term physical disabilities, was identified as a challenge by more than half of the cancer survivors (53.4%). Managing side effects was the most commonly identified challenge for survivors (39.7% of all coded segments). Sub-codes within the managing the side effects domain included physical (26.0%) and functional side effects/sequelae (13.7%).

#### 3.3.1. Physical

Survivors identified a range of physical health challenges often described with technical, medicalized language. One survivor described their greatest challenge as the “ongoing affects from thyroid suppression changing levels. Have issues absorbing meds at times also affects from [radioactive] iodine. My teeth are insanely sensitive post tx” (PID 63). Some survivors were concise in identifying the side effect which was their greatest challenge, such as this survivor who responded “Erectile Dysfunction” (PID 1417). Chronic pain was identified as an issue for survivors, which one survivor described as the “constant pain in my rectum. From the time I come home after my surgery. No one has been able to see or test, also to see what causes that pain-think nerve damage” (PID 487). Other long-term side effects identified by survivors were relatively diverse, with survivors describing experiences such as “neuropathy in feet and right hand” (PID 68). Many survivors noted two or more side effects, which were clearly connected for the survivor as part of the greatest challenge of their cancer. For example, one survivor described side effects of “mobility in R+ arm and dealing with lymphedema” (PID 1189). Another survivor related several side effects of various kinds, writing, “nerve damage in my right shoulder neck. I have extreme dry mouth + teeth breakage all the time. I have a huge hump on my head and I can’t grow hair” (PID 93). Other survivors focused on the experiential aspects of these side effects, such as one survivor who was challenged by the “condition of my body, out come body shape and size, missing ribs” (PID 1693).

#### 3.3.2. Functional

Managing side effects also included limitations on survivors’ abilities to engage in a wide range of meaningful activities. Survivors reported broad, ongoing side effects, such as one who described, “Basically being physically limited due to the effects of cancer and other issues that have happened due to past treatments, has impacted many of the things I am able to do” (PID 800). Another survivor explained, “It’s been a struggle I have a lot of issues since the surgery that have limited me in a lot of ways,” (PID 125) which he directly connected to his ability to contribute to the economic well-being of the household. He continued, “It’s been hard to cope with in many ways because I see my wife work many hours to help try to keep us afloat [and] I should be helping and I can’t” (PID 125).

Some survivors had specific side effects which they described as functionally limiting, such as one survivor who stated, “Some of my GI symptoms make it challenging to get out much” (PID 1152). Another specific functional challenge was side effects related to fertility. One survivor described her experience with “being diagnosed w/Breast Cancer at age 31 (not long after I was married) limited my (our) ability to try to have children. By the time it was ‘safe’ to try (stopping early Tamoxifen) we were never successful” (PID 2059). This survivor expanded on the psychological impact of infertility, writing it “was the hardest emotional journey. We could not afford to freeze eggs before Tamoxifen” (PID 2059).

### 3.4. Managing the economic impact

Approximately one in ten cancer survivors described managing the economic impacts from their cancer and its treatment as one of their biggest challenges (9.3%). Emergent sub-codes in this domain include employment (2.6% of all coded segments), healthcare costs (2.8% of all coded segments), and transportation (1.5% of all coded segments).

#### 3.4.1. Employment

Some survivors experienced challenges related to their employment, including the ability to perform one’s job duties, and loss of employment as an outcome of their cancer or its treatment. Some survivors succinctly stated the challenge of “not being able to work” (PID 82). Another wrote, “I can’t work due to the side effects of the chemo” (PID 847). Other survivors were challenged by not being able to retain or return to their job after the end of active treatment. One survivor described the challenge of “not being able to be employed at my past job due to my present health” which they described as “upsetting” (PID 800). Losing one’s job or having to close one’s business due to cancer and/or treatment were also noted as challenges. One survivor wrote they were “medically terminated from my job” (PID 1149). Another survivor described far-reaching economic effects for themselves and their partner: “I had to sell my business that I spent my entire adult life building up because I was too sick to manage it. My wife is a teacher and should’ve retired when she got to year 28. She’s still working because of my cancer and needing the money for making ends meet” (PID 2660).

#### 3.4.2. Healthcare costs

Survivors described financial challenges related to the affordability of healthcare, generally, and cancer care, specifically. One survivor wrote about the challenge of carrying private insurance but still receiving “big bills” for their care despite carrying private insurance and that “Medicare/Medicaid [patients] are not out as much” (PID 3077). This survivor also described working “2 jobs to get by” and concluded, “I feel the prices of medical care are outrageous” (PID 3077). One survivor described how fears of high healthcare costs provoke and intensify their fear of recurrence, writing:

Sometimes I worry that it may come back even after so many years I’ve been cancer free, or I get a diagnosis with other types of cancers and that will make it difficult for my family to face the financial burden. I did not have this problem with my first diagnosis with Thyroid cancer at the age of 16, because I was still living in Italy and the treatments were almost all free. I just had to worry about my outcome which is already a lot to worry about.

(PID 1904)

Other survivors noted the ongoing costs of survivorship care. One survivor described their biggest challenge as “the constant expenses of check-ups. The surgery was expensive and the check-ups add up” (PID 2065). Medication costs were also noted as a challenge, which one survivor articulated as “taking medicine daily + insurance not covering the brand my Dr. prescribes” (PID 2857). At other times, social isolation with a concomitant reduction in access to shared or community financial supports were challenges for survivors. One survivor described this, stating, “Only myself to rely on. I carry the financial burden” (PID 5569).

#### 3.4.3. Transportation

Access to transportation and the cost of transportation were cited as a financial challenge for cancer survivors. Some survivors simply stated their challenge was “transportation (gas)” (PID 208). For others, transportation was one challenge among several they faced, such as one survivor whose answer ranged across multiple domains: “Physical + mental pain. Lack of means. Support, need transportation. Acceptance” (PID 3399). Other survivors noted transportation challenges related to their current location along the cancer care continuum, such as this survivor who described their largest challenge as the “ongoing evaluation to monitor status” which included the added challenge of “having to travel to Little Rock [urban state capital] for this” (PID 728). At times, transportation challenges may include other mobility and economic issues, as for this survivor who wrote their greatest challenges were “getting to appointments and to appt. sights, parking and have to pay for valet. I have limited mobility” (PID 5663).

Confirmatory analysis of the frequency of themes between survivors with RUCC codes for metropolitan residences (n = 313) and for rural residences (n = 205), with one survivor’s residence not collected, demonstrates relative consonance in cancer survivors’ greatest challenges across the urban-rural continuum. We present the code frequencies in Table 4.

## 4. Discussion

Cancer survivors face numerous challenges stemming from cancer and/or its treatment. Our analysis provides novel empirical data on challenges faced by a diverse group of cancer survivors who received cancer care and primarily resided in Arkansas, a largely rural state. In this study, we used the QoS framework to holistically explore challenges faced by cancer survivors. This is an important contribution, as most research on challenges related to cancer survivorship does not integrate theoretical frameworks to guide the interpretation of findings or facilitate comparison across diverse groups of survivors (Thompson et al., 2024). We identified emergent themes across the four domains of the QoS: quality of life, survival, managing the side effects, managing the economic impact. Of note is our finding that there were no major differences in the challenges reported between survivors living in rural or urban areas. One explanation would be the proximity of rural residence to RUCC-defined urban areas and the rapid growth of suburban and ex-urban areas which has led to the expansion of the definition of areas which still face rural-specific problems. This finding supports the prior literature examining urban-rural differences which has shown that despite designation as metropolitan or urban, the facts on the ground are that the infrastructural and social advantages of metropolitan areas have not yet made an impact on the challenges and daily experiences of cancer survivors living in many of these areas. In other words, areas officially designated as metropolitan in Arkansas and other rural states are often experienced by residents as disconnected and cut off from many central institutions and resources that could promote a healthier and higher quality cancer survivorship experience.

Within quality of life, we found survivors frequently faced challenges relating to the psychological, emotional, and social effects of their cancer, with 78.6% of survivors describing at least one challenge affecting their quality of life. This is broadly consistent with prior literature (Gallicchio et al., 2021). Changes in personality, intense feelings of anxiety, poor self-esteem, guilt, and shame were among the psychological and emotional effects described by survivors. Fear of recurrence was identified as a challenge for many survivors, sometimes accompanied by hypervigilance and anxiety about symptoms of recurrence or related to annual or semi-annual screenings (Simonelli, Siegel, & Duffy, 2017). This is consistent with quantitative studies on the fear of cancer recurrence (Kirsch et al., 2024; Luigjes-Huizer et al., 2022). While a number of quantitative measures for patient-reported outcomes have been developed to measure survivors’ quality of life (Avis et al., 2005; Cella & Stone, 2015; Firkins, Hansen, Driessnack, & Dieckmann, 2020), there has been little focus to date on understanding the quality of survivorship among specific populations at higher risk for healthcare disparities, e.g., underserved rural communities and marginalized racial and ethnic communities (Hallgren, Yeary, et al., 2023; Stout et al., 2024).

Furthermore, there has been limited qualitative research on the challenges faced by survivors in having a high quality of life (Laidsaar-Powell et al., 2019). Thus, our qualitative analysis of a diverse and largely rural population helps fill a critical gap in the survivorship literature especially for cancer survivors’ quality of life in rural areas. Survivors also reported related challenges in their social lives, including reduced ability to engage in meaningful and enjoyable social connections, changes to their social identity related to survivorship, and impacts on sexual intimacy and/or romantic relationships. While the social context of survivorship has been recognized in prior literature, this study is among the few studies to include a holistic focus on the effects of cancer and its treatment on social and emotional well-being and health-related quality of life among diverse survivors diagnosed with a range of cancer types affecting a variety of body systems (Keesing, McNamara, & Rosenwax, 2015; Laidsaar-Powell et al., 2019).

Survivors described challenges in their cancer care, including gaps in delivery of care and in maintaining continuity of care in survivorship. This is consistent with prior quantitative studies in the literature on survivorship (Ratnapradipa et al., 2022; Stout et al., 2024). Limited evidence suggests that patients and family physicians prefer shared care with cancer specialists guided by a survivorship care plan (Mayer, Gerstel, Leak, & Smith, 2012). While patient experiences of care and the large number of measures of patient-reported outcomes, including satisfaction with their treatment, have been a major focus of quantitative research (Grunfeld et al., 1999; Murchie et al., 2010; Vos, Wieldraaijer, van Weert, & van Asselt, 2021), our study is unique and serves to fill the gap in theory-driven, qualitative survivorship research (Thompson et al., 2024).

Among all domains of the QoS framework, survival was the least frequently mentioned by survivors. Only 8.1% of survivors described their biggest challenge as surviving cancer and/or its treatment. Our findings may indicate survivors are not primarily focused on the length of their life after diagnosis, even as life-extension is identified as a primary focus among oncologists and other cancer healthcare providers (Shrestha et al., 2019). One possible explanation for this finding may be that survivors in this study received diagnoses among a wide range of cancer types and locations and included individuals without respect to the stage of their cancer at diagnosis. Thus, some survivors were unlikely to perceive death as a likely outcome of their diagnosis, e.g., for early-stage melanomas or cancers successfully treated through minor surgical interventions. These findings also relate to the wider literature which demonstrates survivors and providers may have different goals for cancer treatment with providers often focused on life extension at the expense of quality of life compared to survivors who may prioritize the quality of their survival over life extension at any cost (Bernacki, Block, & for the American College of Physicians High Value Care Task Force, 2014; W. S. Chou et al., 2017; W.-Y. S. Chou et al., 2022; Udelsman et al., 2019). However, more work is needed to bridge the gap between the health system and provider focus on measurable biological outcomes (e.g., length of survival after diagnosis) and the experiential quality of that survival as experienced and described by survivors themselves (Shrestha et al., 2019).

The management of side effects and sequelae of cancer and its treatment were reported as major challenges for survivors. While most survivors focused on challenging physical and biological symptoms and side effects, loss of ability to engage in everyday activities was also reported as challenging for survivors. This is largely supported in the literature, where managing the side effects of cancer and its treatment are a major concern for survivors (Emery et al., 2022), especially in survivorship care following active treatment (Given & Given, 2013). Our qualitative analysis of a large, diverse sample of survivors across a large variety of cancer locations and types is a novel contribution to the literature, with much of the extant literature focused on specific cancer types or sub-populations of survivors (Laidsaar-Powell et al., 2019).

Economic and financial impacts from cancer diagnosis and treatment were reported challenges for one in ten cancer survivors, including effects on their employment or ability to work, dealing with the costs of cancer treatment, and transportation. This is consistent with prior studies emphasizing the financial toxicity experienced by survivors (Abrams et al., 2021; Firkins et al., 2020; Hallgren, Ayers, et al., 2023; Hallgren et al., 2020; Hallgren et al., 2024; Hallgren, Thompson, et al., 2023; Ponce, Thomas, & Diaz, 2022). While it is unclear why economic impacts were not more frequently identified as challenges by survivors, this may be attributed to the phrasing of the question, which prompted survivors to describe only their “biggest” challenge. There may also be a survivorship bias as large socioeconomic and racial disparities in mortality are a recurring concern raised in the literature (Abrams et al., 2021; Shaw et al., 2024; Sosa et al., 2021; Ward et al., 2004). Additionally, the overwhelming majority of our participants reported they were insured at the time of diagnosis, with access to care facilitated by their coverage.

One-size-fits-all survivorship care has been criticized for the tendency to be medically-focused, centered on clinical care, and for ignoring survivors’ diverse and context-dependent psychosocial needs in the wider social ecology of survivorship (Alfano, Jefford, Maher, Birken, & Mayer, 2019; Stout et al., 2024). While the QoS framework mitigates these issues, it is not without its own limitations. The QoS was developed based on exploratory research with patients with only two cancer types: metastatic non-small cell lung cancer and metastatic melanoma (Fallowfield et al., 2017). As this is the first study to leverage the QoS to analyze challenges among a sample of survivors of a broad range of cancer types and treatment trajectories, we found that the domains of the QoS, particularly quality of life, were not fully operationalized and required further elaboration. Quality of life in particular has been criticized for lacking clear definition or serving as a catch-all for all aspects of survivorship (Dijkers, 1999). There is also a lack of consensus among researchers as to what constructs are included within quality of life and a “truly bewildering range of contents and measurement approaches” according to Marcel Dijkers (Dijkers, 1999). These range from generic quantitative instruments (e.g., the Health-Related Quality of Life [HRQoL] and Short Form 36 [SF-36]) (Fallowfield, 2009), cancer-specific instruments (e.g., the European Organisation for Research and Treatment of Cancer [EORTC] QLQ-C30 and Functional Assessment of Cancer Therapy-General [FACT-G]), and measures focused on specific populations or among patients with specific cancers (e.g., the Quality of Life in Adult Cancer Survivors [QLACS] or the National Comprehensive Cancer Network-Functional Assessment of Cancer Therapy-Breast Cancer Symptom Index-16 [NFBSI-16]).

Multidimensional patient-reported outcomes have also been employed in problematic ways, e.g., measured by instruments which have not been psychometrically validated or which do not assess quality of life holistically (Dijkers, 1999; Fallowfield, 2009). The incredible profusion of quality of life measures also creates major issues in the collection, analysis, and comparison across studies (Firkins et al., 2020). Additionally, comprehensive multidimensional patient-reported outcomes tend to be exclusively quantitative, thus providing thin descriptive value of key constructs, limited flexibility of response, and superficial assessment of complex constructs (Paltridge & Phakiti, 2010), with recent calls to expand patient-reported outcomes to include qualitative and narrative approaches to enhance our understanding and interpretation of this data (K. Meadows, 2021; K. A. Meadows & Reaney, 2023). The present study is the first study to demonstrate the utility of the QoS as a qualitative approach to understanding quality of life in the context of a holistic quality of survival. Qualitative and mixed-methods approaches to understanding the cancer survivors’ quality of survival can inform evidence-based approaches to enhancing survivor well-being through timely, targeted intervention (Gallicchio et al., 2021). This may also inform gaps in health sciences and continuing education curricula to better align provider and survivor goals in cancer care. Providing cancer care providers with holistic tools to conceptualize cancer patients’ lived experiences can have direct applications in care delivery tailored to specific types of patients or for training in specific patient-centered techniques, such as shared decision-making in cancer care (Elkefi & Asan, 2024; Hakim et al., 2024).

These results also highlight some areas of concern within the QoS framework, if it is to be leveraged to understand the lived experience of cancer survivors and the production of qualitative or mixed-methods datasets of patient-reported outcomes. Particularly, constructs in the QoS required significant discussion among the research team to accurately code differences between closely-related concepts (e.g., fear of recurrence vs. survival). Ultimately, the research team approached the problem of parsimony through an iterative process delineating participants’ challenges related to the length of their life, not dying, or staying alive (survival) compared to challenges emerging from psychosocial processes (e.g., emotional or affective states such as anxiety or fear). However, the lack of extant methodological research focused on these issues highlights the urgent need for further refinement of these core constructs to ensure the reliability and utility of patient-reported outcomes or measures, whether generic or specific in focus. The lack of elaboration and operationalization of these issues within the QoS, then, must be understood in the wider context of patient-reported outcomes, generic or specific to survivorship, which have frequently been similarly critiqued (Adeghe, Okolo, & Ojeyinka, 2024; Pangestu & Rencz, 2023; Rothmund, Meryk, et al., 2023; Rothmund, Sodergren, et al., 2023). Future research needs to be conducted to resolve these methodological and conceptual issues to deliver on the promise of these instruments for improving healthcare and well-being of patients, especially for cancer survivors (Adeghe et al., 2024; Carreira et al., 2021; Churruca et al., 2021; Dijkers, 1999; Rothmund, Meryk, et al., 2023).

### 4.1. Strengths and limitations

This study leverages an exploratory qualitative approach to understand challenges faced by cancer survivors in Arkansas, facilitating holistic analysis of the lived experiences of these survivors in their own words. This study is not without limitations, however. While our sample of cancer survivors was diverse in cancer type, the sample was drawn from patients diagnosed and/or treated for cancer in Arkansas, which may limit the transferability of our findings to survivors in urban areas, in other regions of the US, or to international populations. Second, due to the broad inclusion criteria, these findings may not be indicative of challenges faced by survivors of specific cancers/cancer sites (e.g., colorectal, hematological, dermatological, etc.) or those who received specific treatments (e.g., survivors receiving various types of chemotherapy versus out-patient surgical interventions). Additionally, we excluded potential participants with recent diagnoses which can have effects on the applicability of our findings to individuals who are undergoing diagnosis or are recently diagnosed. Our results, therefore, must be understood as fully comprehensive of all survivors, and our results may not reflect the changes in survivors’ greatest challenges faced as they move along the cancer care continuum.

Despite these limitations, this qualitative, theory-driven study included a large, diverse sample of survivors experiencing a range of cancer types and locations which can be used to inform future holistic, pragmatic interventions focused on comprehensive cancer care and quality of life in survivorship irrespective of location or type of cancer.

## 5. Conclusion

Overall, survivors faced a range of challenges with their cancer and its treatment including challenges with their quality of life, survival, managing side effects, and managing the economic impact of cancer. While our findings are consistent with extant literature, survivorship research has primarily focused on narrowly-defined, quantitative measures of patient-reported outcomes which do not provide holistic insight into the lived experiences of cancer survivors. Much of the literature also lacks grounding in a broader theoretical understanding of survivorship. This can limit our ability to compare measures across studies that may lack conceptual clarity and/or operationalize key constructs in drastically different ways. Our analysis of the challenges faced by a diverse sample of cancer survivors highlights the importance of understanding the psychosocial challenges and physical sequelae of cancer and its treatment, which were frequently identified as the biggest challenges facing survivors. Our research helps fill the significant gaps in the literature, improving our understanding of the sequelae faced by cancer survivors in rural communities. Nuanced understanding of survivors’ experiences, which uses theory-driven qualitative and mixed-methods approaches, will continue to be critical for developing effective evidence-based practices which meet the needs of diverse communities of survivors. This research will be used in the development of clinical and pedagogical interventions to support cancer survivors and can inform future research and novel interventions for survivors in rural communities and across the US.

## Figures and Tables

**Table 1:** Sociodemographics (n = 519).

Category	Freq (%)^a^	Mean	Range
Age (n = 508)^b^		63	21–95
Rurality (n = 518)^b^			
Metropolitan/urban	312 (60.2%)		
Nonmetropolitan/rural	206 (39.8%)		
Gender (n = 517)^b^		3.75	
Man	204 (39.5%)		
Woman	311 (60.2%)		
Transgender	1 (0.2%)		
Gender non-conforming	1 (0.2%)		
Race/ethnicity (n = 509)^b^			
Asian	4 (0.8%)		
Black or African American	74 (14.5%)		
Hispanic	10 (2.0%)		
Native American/Alaska Native	9 (1.8%)		
White	412 (80.9%)		
Relationship status (n = 515)^b^			
Single, never married	40 (7.8%)		
Married or co-habitating	320 (62.1%)		
Separated/divorced/widowed	155 (30.1%)		
Current employment (n = 514)^b^			
Disabled	85 (16.5%)		
Employed	148 (28.8%)		
Retired	223 (43.4%)		
Unemployed	30 (5.8%)		
Other	28 (5.4%)		
Education (n = 513)^b^			
Less than high school	29 (5.7%)		
High school graduate	100 (19.5%)		
Vocational/technical post-HS	55 (10.7%)		
Some college, no degree	116 (22.6%)		
College graduate	127 (24.8%)		
Postgraduate	86 (16.8%)		
Insurance coverage, at diagnosis (n = 496)^b^			
Yes	488 (98.4%)		
No	8 (1.6%)		

HS = high school.

a Percentages may not total 100% due to rounding.

b Valid responses.

**Table 2:** Cancer site or type.

Site/type^a^	Freq (%)
Bladder	13 (2.5%)
Bone	3 (0.6%)
Brain	10 (1.9%)
Breast	81 (16.0%)
Cervical	15 (2.9%)
Colon	34 (6.6%)
Endometrial	28 (5.4%)
Esophageal	2 (0.4%)
Head and neck	34 (6.6%)
Heart	1 (0.2%)
Hodgkin’s Lymphoma	8 (1.5%)
Larynx	2 (0.4%)
Leukemia	25 (4.8%)
Liver	7 (1.4%)
Lung	34 (6.6%)
Melanoma	36 (6.9%)
Non-Hodgkin’s Lymphoma	22 (4.2%)
Oral	2 (0.4%)
Other skin cancer	45 (8.7%)
Ovarian	13 (2.5%)
Pancreatic	3 (0.6%)
Pharyngeal	9 (1.7%)
Prostate	32 (6.2%)
Rectal	15 (2.9%)
Renal	22 (4.2%)
Stomach	7 (1.4%)
Testicular	4 (0.8%)
Thyroid	23 (4.4%)

a Participants could select multiple cancer types. Thus, total number of responses is higher than sample size.

**Table 3:** Qualitative codes and frequencies.

Codes & sub-codes	% of coded segments^a^	% of survivors^b^
Quality of life		78.6%
Psychological/emotional	28.2%	
Social	5.4%	
Ongoing/follow-up care	14.8%	
TOTAL	**48.4%**	
Survival		8.1%
Survival	4.9%	
TOTAL	**4.9%**	
Managing the side effects		53.4%
Physical	26.0%	
Functional	13.7%	
TOTAL	**39.7%**	
Managing the economic impact		9.3%
Employment	2.6%	
Healthcare costs	2.8%	
Transportation	1.5%	
TOTAL	**6.9%**	

a Percentages may not total 100% due to rounding.

b Percentages do not total 100% due to code co-occurrence within valid responses.

**Table 4:** Geographic comparison of qualitative codes and frequencies.

Codes & sub-codes	% of coded segments^a^	
	Metro	Rural
Quality of life		
Psychological/emotional	26.4%	28.5%
Social	3.5%	7.7%
Ongoing/follow-up care	7.1%	9.5%
TOTAL	**37.0%**	**45.6%**
Survival		
Survival	16.1%	11.7%
TOTAL	**16.1%**	**11.7%**
Managing the side effects		
Physical	27.2%	22.3%
Functional	12.8%	13.9%
TOTAL	**40.1%**	**36.1%**
Managing the economic impact		
Employment	2.5%	2.6%
Healthcare costs	2.8%	2.6%
Transportation	1.5%	1.5%
TOTAL	**6.8%**	**6.6%**

a Percentages may not total 100% due to rounding.

## Data Availability

The deidentified data underlying the results presented in this study may be made available upon reasonable request from the corresponding author, Dr. Emily Hallgren, at emily.hallgren@med.uvm.edu. The data are not publicly available in accordance with funding requirements and participant privacy.
